# Supplementary material for: Evolution of nanopores in hexagonal boron nitride
Source: Commun Chem. 2023 Jun 5;6:108. doi: 10.1038/s42004-023-00899-1 (PMC10241886; doi:10.1038/s42004-023-00899-1)
Supplement: Supplementary file 2 — Supplemental Information [file 42004_2023_899_MOESM2_ESM.pdf]

Supplementary Materials

Evolution of Nanopores in Hexagonal Boron Nitride

Chunhui Dai[1,2,3], Derek Popple[2,3,4], Cong Su[1,2,3], Ji-Hoon Park[5], Kenji Watanabe[6], Takashi Taniguchi[6], Jing Kong[5], Alex Zettl[1,2,3*]

1. Department of Physics, University of California at Berkeley, Berkeley, CA 94720, USA.
2. Materials Sciences Division, Lawrence Berkeley National Laboratory, Berkeley, CA 94720, USA
3. Kavli Energy NanoSciences Institute at the University of California Berkeley and the Lawrence Berkeley National Laboratory, Berkeley, CA 94720, USA.
4. Department of Chemistry, University of California at Berkeley, Berkeley, CA 94720, USA'
5. Department of Electrical Engineering and Computer Science, Massachusetts Institute of Technology, Cambridge, MA, USA
6. International Centre for Materials Nanoarchitectonics, National Institute for Materials Science, Tsukuba, Japan

*Corresponding author. Email: azettl@berkeley.edu

TEM Characterization of Monolayer Hexagonal Boron Nitride (h-BN) Membrane:

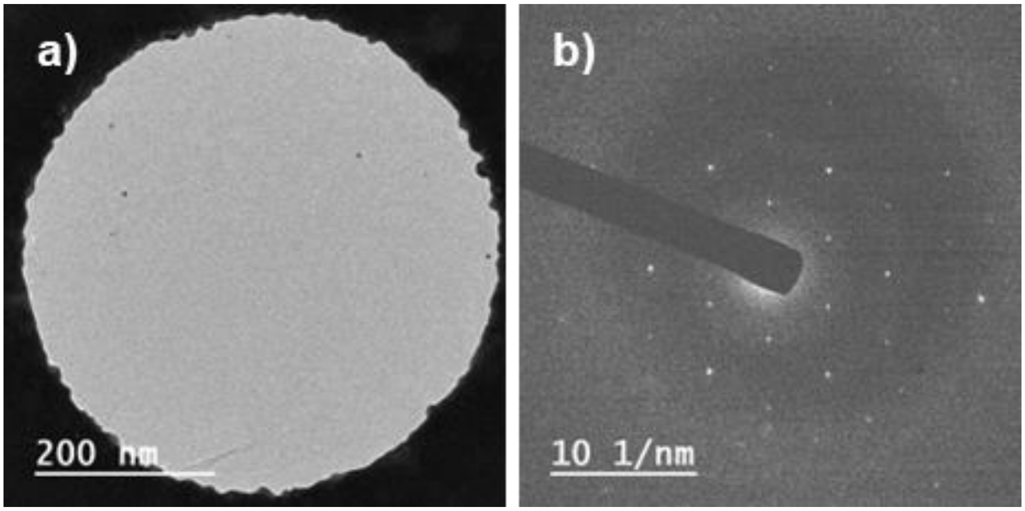

Figure S1, a) TEM image of a monolayer h-BN membrane transferred onto a holey TEM grid through a wet transfer process. b) Selected area electron diffraction (SEAD) of the monolayer h-BN studied in this work.

Time Series of Defects form in Bernal Stacked Mulatilayer h-BN:

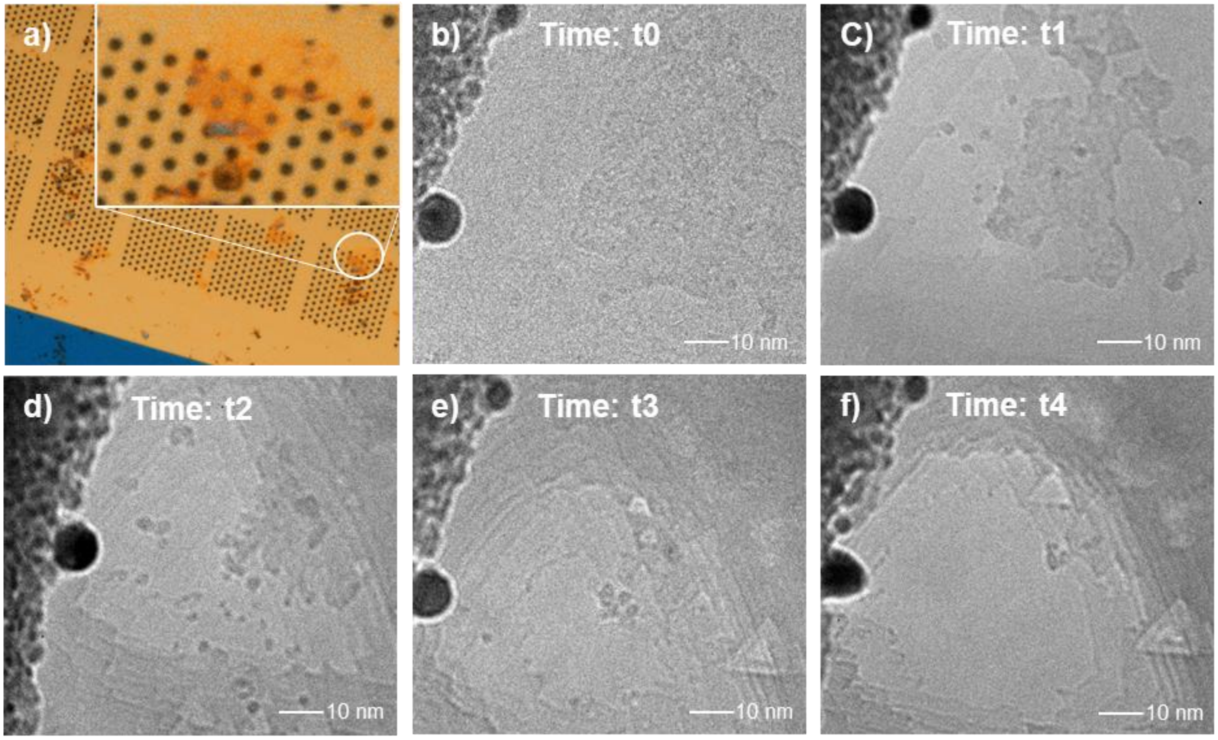

Figure S2, a) Multilayer Bernal stacked h-BN transferred onto a holey TEM grid through a wet transfer process. The membrane is ~10nm. b) TEM image of the initial condication (Time: t0) of the membrane. c-f) TEM time series (Time: t1 to t4) showing the formation of nanopores and stripping of layers under an electron beam with a beam current of ~40 A/cm^2. The time interval between tn and tn+1 is approximately 30 minutes. The beam is expended for imaging with a beam current of ~3 A/cm^2.
